# Alzheimer’s Disease and Specialized Pro-Resolving Lipid Mediators: Do MaR1, RvD1, and NPD1 Show Promise for Prevention and Treatment?

**DOI:** 10.3390/ijms21165783

**Published:** 2020-08-12

**Authors:** Keishi Miyazawa, Hisanori Fukunaga, Yasuko Tatewaki, Yumi Takano, Shuzo Yamamoto, Tatsushi Mutoh, Yasuyuki Taki

**Affiliations:** Department of Nuclear Medicine and Radiology, Institute of Development, Aging and Cancer, Tohoku University, Sendai 980-8575, Japan; keishi.miyazawa.p6@dc.tohoku.ac.jp (K.M.); yumi.takano.b7@tohoku.ac.jp (Y.T.); shuzo.yamamoto.c7@tohoku.ac.jp (S.Y.); tmutoh@tohoku.ac.jp (T.M.); yasuyuki.taki.c7@tohoku.ac.jp (Y.T.)

**Keywords:** Alzheimer’s disease, neuroinflammation, resolution of inflammation, SPMs, MaR1, RvD1, NPD1, novel approaches

## Abstract

Alzheimer’s disease (AD) is a common neurodegenerative disease and a major contributor to progressive cognitive impairment in an aging society. As the pathophysiology of AD involves chronic neuroinflammation, the resolution of inflammation and the group of lipid mediators that actively regulate it—i.e., specialized pro-resolving lipid mediators (SPMs)—attracted attention in recent years as therapeutic targets. This review focuses on the following three specific SPMs and summarizes their relationships to AD, as they were shown to effectively address and reduce the risk of AD-related neuroinflammation: maresin 1 (MaR1), resolvin D1 (RvD1), and neuroprotectin D1 (NPD1). These three SPMs are metabolites of docosahexaenoic acid (DHA), which is contained in fish oils and is thus easily available to the public. They are expected to become incorporated into promising avenues for preventing and treating AD in the future.

## 1. Introduction

Alzheimer’s disease (AD) is currently the most common cause of dementia [1]. While cholinesterase inhibitors and N-methyl-D-aspartate (NMDA) receptor antagonists are typically used to attenuate the symptoms and progression of dementia, they do not provide complete treatment nor do any other therapies, till date. Thus, more and more people are developing AD all over the world and obviously it has become a global issue. Hence, the urgent need to stem the increasing prevalence of AD [2] has prompted the improvement of such mainstay prevention and treatment strategies.

Characterized by the deposition of amyloid-β (Aβ) outside neurons [3,4,5,6,7,8] and hyperphosphorylated tau proteins within [9], the pathology of AD is largely ascribed to the activation of immune system, such as microglia [10,11,12], which leads to neuroinflammation induced by the disruption of hypothalamic-pituitary-adrenal hormonal homeostasis by various chronic stress factors [13], including infection, invasive injury, and autoimmune response [14,15,16,17,18,19,20]. In the central nervous system (CNS), glial cells such as oligodendroglia, microglia, and astrocytes play an important role in mediating neuroinflammation by maintaining the balance between extracellular ions and neurotransmitters [21]. Aβ activates microglia and astrocytes, causing chronic inflammation [15].

Attempts to prevent the onset of AD with anti-inflammatory drugs found that the long-term treatment of rheumatoid arthritis with non-steroidal anti-inflammatory drugs (NSAIDs) reduced the incidence of AD [22,23,24,25,26] and that intranasal steroids are effective in mitigating the symptoms of patients with the early stages of AD and olfactory impairment [27]. However, systematic review has not found NSAIDs to be effective in randomized controlled trials, and NSAIDs are not currently recommended for the treatment of AD [28,29].

While most of the previous research on inflammation has focused on inhibiting the synthesis of inflammatory substances, the goal of more recent research has shifted to the resolution of inflammation. More specifically, the pathway seems to follow this order: inflammatory substances are synthesized to produce inflammatory lipid mediators, prostaglandins (PGs) and thromboxanes (TXs) via cyclooxygenase enzymes (COXs), and leukotrienes (LTs) via lipoxygenase enzymes (LOXs), by arachidonic acid (AA), one of the ω-6 polyunsaturated fatty acids (ω-6 PUFAs) [30,31,32,33,34]. PGs and LTs are well-known pro-inflammatory lipid mediators that induce fever and pain. As anti-inflammatory drugs, NSAIDs inhibit the activity of COXs. While inflammation is considered to be initiated by an active mechanism, its resolution had been thought of as a passive process. Once lipoxin A4 (LXA4), which is produced from AA by 15-LOX and 5-LOX, was reported to have an anti-inflammatory effect [35,36,37], increasingly more research has focused on the resolution of inflammation. Specialized pro-resolving lipid mediators (SPMs) are a group of lipid mediators that resolve inflammation [38]; they include the ω-3 polyunsaturated fatty acid (ω-3 PUFA) metabolites of docosahexaenoic acid (DHA) and eicosapentaenoic acid (EPA) contained in fish oils [39], which are easily available to the public. DHA is metabolized by 15-LOX and 5-LOX into either maresin 1 (MaR1), resolvin D1 (RvD1), or neuroprotectin D1 (NPD1) [40,41]. EPA is metabolized into resolvin E1 (RvE1) by CYP450 or 15-LOX [42]. These SPMs are produced in human tissue and resolve inflammation [43]. It is called resolution of inflammation [43,44,45,46,47,48].

Recent studies identified receptors for SPMs in the human brain, reduced levels of SPMs in the brains of patients with AD, and a correlation between lower SPM levels and cognitive impairment [49]. Furthermore, SPMs were shown to promote neuronal survival and increase the microglial phagocytosis of Aβ [48,50,51]. The pathways that produce PGs and LTs from AA are aberrantly activated in patients with AD [52,53], and lesser degrees of cognitive impairment among older adults were associated with higher blood concentrations of ω-3 PUFAs [54]; SPMs might be involved in both findings.

The potential use of SPMs to resolve inflammation could be a promising approach to the treatment of AD. The purpose of this review is to compile knowledge concerning the mechanistic role of SPMs in chronic neuroinflammation associated with AD and to summarize future research strategies for novel therapies using SPMs. In addition to an overview of SPMs in general, we focus on three produced in the human brain: MaR1, RvD1, and NPD1 [50,55,56].

## 2. SPMs (Specialized Pro-resolving Lipid Mediators)

SPMs belong to a superfamily of metabolites derived from ω-3 and ω-6 PUFAs that actively resolve inflammation and promote the return to homeostasis by limiting inflammatory signals and suppressing the natural response during inflammation [57,58,59,60]. Examples of ω-6 PUFAs include AA, which is used by COXs to synthesize PGs and by LOXs to synthesize LTs. These lipid mediators participate in the AA cascade and cause inflammation. However, LXA4, which is synthesized from AA via LOXs, is an SPM that exerts anti-inflammatory effects. The ω-3 PUFAs include DHA and EPA, which are abundant in fish oils [39]. The enzyme induced by fat-1 gene converts ω-6 PUFAs to ω-3 PUFAs, and experiments in mice transfected with this gene showed that lipid mediators, not other ingredients contained in food, not only stimulate inflammation but also help to resolve inflammation [61]: i.e., decrease the levels of pro-inflammatory cytokines and increase the levels of anti-inflammatory cytokines [62]. The discovery of LXA4 revealed that inflammation can be actively resolved through SPMs [35,36,37]. SPMs stimulate physiological signals to resolve and end inflammation and return to physiologically normal conditions [48], whereas it is man-made, not physiological that anti-inflammatory drugs such as NSAIDs block the production of pro-inflammatory chemicals.

**Figure 1:**
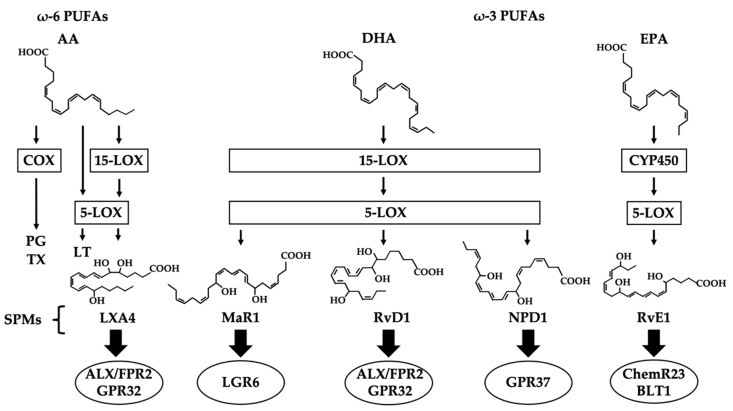
Pathways of SPM synthesis.

Four types of SPMs involved in AD were identified: LXA4, produced from AA by 15-LOX and 5-LOX; MaR1, RvD1 and NPD1, produced from DHA by 15-LOX and 5-LOX; and resolvin E1 (RvE1), produced from EPA by CYP450 and 5-LOX [58,63,64,65]. While the receptors for LXA4, RvD1, and RvE1 were identified, those for MaR1 and NPD1 were not. SPM receptors belong to the 7-transmembrane G protein-coupled receptor (GPCR) [66]. LXA4 and RvD1 bind to LXA4/formyl peptide receptor 2 (ALX/FPR2) and G protein receptor (GPR) 32 [67,68,69,70]. ALX/FPR2 is also a receptor for Aβ, which functions as a ligand that activates microglia and transmitting pro-inflammatory signals [71]. RvE1 binds to chemerin receptor 23 (ChemR23) and the leukotriene B4 receptor 1 (BLT1) [72]. Leucine-rich repeat containing G protein–coupled receptor 6 (LGR6) and G protein–coupled receptor (GPR37) are thought to be possible receptors for MaR1 and NPD1, respectively [73,74,75,76,77] (Figure 1). Since pro-inflammatory ligands aside from SPMs also bind to these receptors [71,78,79], very low concentrations of SPMs can bind to their receptors and signal the resolution of inflammation [77]. A nuclear receptor with anti-inflammatory function, peroxisome proliferator-activated receptor (PPAR)-γ is also a receptor for SPMs such as LXA4, RvD1, and NPD1 that mediates neuroprotective effects in neurons [51,80,81]. PPAR-γ agonists reportedly increase the level of LXA4, and PPAR-γ inhibitors were shown to reduce the level of LXA4 [82]. Retinoic acid-related orphan receptor α (RORα) is also a nuclear receptor with anti-inflammatory function and is thought to be a receptor for MaR1 [74,75].

SPMs inhibit immune cell infiltration, down-regulate pro-inflammatory mediators, up-regulate anti-inflammatory mediators, and promote phagocytosis and tissue repair [50,83,84,85]. The production of pro-inflammatory lipid mediators is class-switched to the production of inflammation-resolving lipid mediators under certain conditions [86,87,88]. Although the detailed mechanism for the class-switching is unclear, this finding indicates that it can be programmed to induce pro-resolving lipid mediators [50]. Moreover, SPMs are detectable in the brain using liquid chromatography mass spectrometry and can be regulated by nutrition such as dietary supplements [89].

Lower levels of LXA4 and NPD1 were reported in patients with severe asthma and decreased concentrations of SPMs were observed in patients with localized aggressive periodontitis relative to healthy controls [90,91,92]. In addition, SPMs inhibit the accumulation of eosinophils and lymphocytes in a mouse model of asthma [93] and inhibit neutrophil infiltration and inflammatory cytokines in a mouse model of sepsis [94]. Furthermore, SPMs reportedly promote the resolution of inflammation in mouse models of peritonitis, enteritis, retinopathy, and inflammatory pain [36,95,96,97].

SPMs repair neurons and promote the microglial phagocytosis of Aβ, changing the microglial phenotype from pro-inflammatory to anti-inflammatory [48]. The levels of LXA4, MaR1, and NPD1 are low in the hippocampi of patients with AD [50], and the concentration of LXA4 is reduced in their cerebrospinal fluid (CSF) [49]. These reductions in SPMs are significant in so far as they present concurrently with increased inflammation. Decreases in LXA4 and RvD1 were correlated with cognitive decline, as measured by scores on the Mini-Mental State Examination (MMSE); however, the detailed mechanism underlying this association remains equivocal [49]. The induction of SPM production in a mouse model of AD promoted the resolution of neuroinflammation, microglial phagocytosis, and memory improvement [98]. Since DHA can cross the blood-brain barrier (BBB) [99], cognitive function could be promoted by administering ω-3 PUFA supplements. Indeed, epidemiological research has shown that the increased intake of ω-3 PUFAs reduces the risk of dementia [100,101,102], while clinical trials in which patients with AD were administered ω-3 PUFAs found that the supplements benefited those with mild cognitive impairment (MCI) but not patients with advanced AD [103,104,105]. This suggests that the factor that metabolizes ω-3 PUFAs into SPMs is deficient in late-stage AD, that the inflammation-resolving effect of SPMs is significant only during the early stages of AD, or that external factors other than ω-3 PUFA pathways are involved such as the ratio of ω-3/ω-6 PUFAs [106]. Although DHA reportedly improves the cognitive impairment of AD patients to a nonsignificant extent [107,108,109], these studies may have been compromised by the nonuniform quality of ω-3 PUFA supplements on the market [110]; larger, more rigorous clinical studies of the potential benefit of DHA in treating AD over longer periods of time are thus warranted. The following sections will focus on MaR1, RvD1, and NPD1, which are produced in the human brain [50,55,56], and discuss the relationship among these SPMs and AD.

## 3. MaR1 (Maresin 1)

MaR1 is produced from DHA by 15-LOX and 5-LOX. The receptor for MaR1 is thought to be LGR6, and RORα of a nuclear receptor [73,74,75]. (Figure 1) MaR1 exerts a potent anti-inflammatory effect and promotes the macrophage phagocytosis and efferocytosis of apoptotic cells [111,112,113]. In addition to prompting the migration of leukocytes, MaR1 also promotes tissue regeneration at the site of injury [111,113]. MaR1 is produced at the late stage of inflammation [114,115,116] and has a potent analgesic effect that controls not only neuropathic pain but also the resolution of local inflammation and its associated pain [111,113] (Table 1).

The level of MaR1 is significantly reduced in the hippocampus and entorhinal cortex of patients with AD [49,50]. MaR1 plays a neuroprotective role in neurons, influencing cell signaling pathways pertinent to inflammatory cell survival, autophagy, axonogenesis, and the inhibition of apoptosis [122,123]. MaR1 up-regulates the level of microglia that are down-regulated by Aβ in the course of AD [122]; in the context of the RvD1- and LXA4-mediated increase in the microglial phagocytosis of Aβ by the action of, this finding suggests that MaR1 plays a distinct role in the microglia-mediated removal of Aβ [50]. These findings suggest that the induction of MaR1 could be a novel, effective approach to treat AD. In model mice, MaR1 treatment decreased the production of pro-inflammatory cytokines, such as TNF-α and IL-6; increased the production of anti-inflammatory cytokines, including IL-2 and IL-10; and mitigated cognitive decline [124].

Furthermore, a prospective human study showed that the intake of DHA, the precursor of MaR1, significantly reduced the incidence of AD [125,126]. On the other hand, there are reports that while DHA treatment improved memory impairment in mice, MaR1 treatment did not [127,128]. Considering that ω-3 PUFAs were effective only in addressing MCI and not advanced AD [103,104,105], it is necessary to elucidate the mechanism by which DHA is metabolized into MaR1 and the biochemical relationship between MaR1 and its receptor.

## 4. RvD1 (Resolvin D1)

RvD1 is produced from DHA by 15-LOX and 5-LOX [114]. The receptors for RvD1 are the same as those for LXA4: ALX/FPR2 and GPR32 [67,68,69,70] (Figure 1). RvD1 effects anti-inflammation by down-regulating pro-inflammatory cytokines, such as IL-1 and IL-6, and up-regulating anti-inflammatory profiles, such as IL-1 antagonists and NF-κB [117,118,129,130]. RvD1 enhances tissue remodeling in microglia by promoting the activation of the signal transducer and activator of transcription 6 (STAT6), as well as PPAR-γ transcriptional factors, through IL-4 activation [119]. (Table 1.)

Murine research has demonstrated that RvD1 improves inflammation-induced cognitive impairment when administered before or during invasive surgery [131,132]. More research on the relationship between RvD1 and AD-related neuroinflammation is ongoing. In vitro, RvD1 promotes macrophage phagocytosis of Aβ and inhibits apoptosis through GPR32 [117,118]. Recent studies showed that RvD1 promotes the phagocytosis of Aβ by modulating MEK1/2, PKA, and PI3K/Akt/mTOR pathways, which are responsible for supporting cell survival upon exposure to oxidative stress [117,133].

Reduced in the CSF of patients with AD, the levels of RvD1 and LXA4 correlate with lower MMSE scores [49]. The increased expression of ALX/FPR2 in patients with AD suggests enhanced neuroinflammation [134]; however, since Aβ is also a ligand for ALX/FPR2 [71], the increased expression of the receptor may either indicate a compensation for reduced concentrations of SPMs or that ALX/FPR2 transmits different signals depending on the site to which a ligand binds. The administration of ω-3 PUFA supplementation, the precursor of RvD1, to patients with MCI increases the levels of RvD1 and enhances the phagocytosis of Aβ, resulting in improved MMSE scores [135].

## 5. NPD1 (Neuroprotection D1)

NPD1 is produced from DHA by 15-LOX and 5-LOX. The receptor for NPD1 is thought to be GPR37 and is PPAR-γ of a nuclear receptor [76,77]. (Figure 1) NPD1 is rapidly synthesized in response to inflammation to promote cell survival [51,136], neuroinflammatory signaling and transcription, and homeostatic regulation [120]. (Table 1).

The levels of NPD1 are significantly reduced in the hippocampi of patients with AD [49,137,138]. NPD1 inhibits apoptosis and promotes cell survival by regulating BCL-2 anti-apoptotic protein and microglia levels [139]. NPD1 suppresses the production of amyloid precursor protein (APP) by the mechanism that activating α-secretase and inhibiting β-secretase through PPAR-γ changes APP from an amyloidogenic pathway into a non-amyloidogenic one, and thus NPD1 effects anti-inflammation [51,120,121]. It has also been suggested that NPD1 may also promote cell survive and regulate the NF-κB cyclic response [140] by stimulating the PI3K/Akt/mTOR pathways [141]. Additionally, in vitro studies showed that APP promotes NPD1 production at concentrations exceeding a specific limit—even in the absence of neuronal DHA [137]—suggesting that APP not only shifts the amyloidogenic pathway to the non-amyloidogenic pathway but also activates 15-LOX, which is involved in NPD1 biosynthetic enzymes [121,142,143]. Although the role of NPD1 in cell signal transduction has received much attention, few clinical studies of AD considered the potential use of NPD1 alone or with ω-3 PUFAs.

## 6. Discussion

As ω-3 PUFAs, the precursor of SPMs, are plentiful in fish oils and readily available to the general public, future developments in the use of ω-3 PUFAs to treat MCI and AD are expected. As SPMs are easily oxidized and converted to other metabolites, it is currently difficult to consider SPMs as a drug target for organs to which they cannot be administered directly. However, there is a great promise for the development of analogs and agonists of SPMs to treat cognitive impairment. Some targeted perspectives for future research are as follows. First, investigating the kinetics of administered DHA after it passes through the BBB will help to determine whether DHA supplements are effective in producing SPMs in the brain and will inform a clearer approach to their application to the prevention and treatment of AD. Second, as few large-scale studies investigated the relationship among AD and SPMs by administering ω-3 PUFAs and DHA, large, long-term clinical studies that use supplements of a uniform quality are warranted. With the accumulation of positive evidence, modulating nutrition could encourage the prevention of AD. Third, since APOEε4 mice reportedly reduced SPM levels [144], research on the relationship among SPMs and APOE, which is highly relevant to AD, could lead to the discovery of a promising prevention strategy. Fourth, although almost all the current methods for measuring neural levels of SPMs are based on the analysis of pathological samples, a noninvasive method of collecting samples with the imaging of magnetic resonance imaging (MRI), positron emission tomography (PET), and single photon emission computed tomography (SPECT) may help to elucidate the dynamics of SPM activity in the brain. Fifth, inhibiting the metabolism of SPMs will provide more anti-inflammatory effects if it is clarified how they are metabolized, even though the metabolic pathways of many lipid mediators are not yet fully understood.

## 7. Conclusions

This review summarizes the latest developments in research on neural MaR1, RvD1 and NPD1: SPMs that promote the resolution of inflammation by down-regulating pro-inflammatory mediators and up-regulating anti-inflammatory mediators. As their mechanisms have yet to be fully explored, understanding their roles in resolution of inflammation is imperative for their application to the development of preventive and therapeutic strategies to address the incidence and progression of AD.

## Figures and Tables

**Table 1:** Bioaction of MaR1, RvD1, and NPD1.

SPMs	Receptor	Bioaction in AD	Reference
MaR1	LGR6	Promote microglial phagocytosis of Aβ Promote tissue regeneration Analgesic effect	[50,111,113]
RvD1	ALX/FPR2 & GPR32	Enhance tissue remodeling in microglia Promote macrophage phagocytosis of Aβ	[117,118,119]
NPD1	GPR37	Influence cell survival, neuroinflammatory signaling and transcription Suppress the production of APP	[51,120,121]

## Abbreviations

AA	Arachidonic acid
AD	Alzheimer’s disease
ALX/FPR	Lipoxin A4/formyl peptide receptor
APP	Amyloid precursor protein
Aβ	Amyloid-beta
BBB	Blood-brain barrier
BLT	Leukotriene B4 receptor
ChemR	Chemerin receptor
CNS	Central nervous system
COX	Cyclooxygenase
CSF	Cerebrospinal fluid
DHA	docosahexaenoic
EPA	eicosapenraenoic
GPCR	G protein-coupled receptor
GPR	G protein receptor
LGR	Leucine-rich repeat containing G protein-coupled receptor
LT	Leukotriene
LOX	Lipoxygenase
LXA4	Lipoxin A4
MaR1	Maresin 1
MCI	Mild cognitive impairment
MMSE	Mini-Mental State Examination
MRI	Magnetic resonance imaging
NMDA	N-methyl-D-aspartate
NPD1	Neuroprotectin D1
NSAIDs	Non-steroidal anti-inflammatory drugs
PET	Positron emission tomography
PG	Prostaglandin
PPAR	Peroxisome proliferator-activated receptor
PUFA	Polyunsaturated fatty acid
RORα	Retinoic acid-related orphan receptor α
RvD1	Resolvin D1
RvE1	Resolvin E1
SPECT	Single photon emission computed tomography
SPM	Specialized pro-resolving lipid mediator
TX	Thromboxane
